# Mechanically Robust, Recyclable, and Self‐Healing Polyimine Networks

**DOI:** 10.1002/advs.202300958

**Published:** 2023-04-23

**Authors:** Ping Yu, Haiyue Wang, Ting Li, Guimei Wang, Zichen Jia, Xinyu Dong, Yang Xu, Qilin Ma, Dongen Zhang, Hongliang Ding, Bin Yu

**Affiliations:** ^1^ School of Environmental and Chemical Engineering Jiangsu Key Laboratory of Function Control Technology for Advanced Materials Jiangsu Ocean University Lianyungang Jiangsu 222005 P. R. China; ^2^ State Key Laboratory of Fire Science University of Science and Technology of China Hefei Anhui 230026 P. R. China; ^3^ Jiangsu Marine Resources Development Institute Lianyungang Jiangsu 222005 P. R. China; ^4^ Shanghai Cedar Composites Technology Co., Ltd 201306 Shanghai P. R. China

**Keywords:** bulky pendant units, copolymerization, polyimine network, thermoset materials

## Abstract

To achieve energy saving and emission reduction goals, recyclable and healable thermoset materials are highly attractive. Polymer copolymerization has been proven to be a critical strategy for preparing high‐performance polymeric materials. However, it remains a huge challenge to develop high‐performance recyclable and healable thermoset materials. Here, polyimine dynamic networks based on two monomers with bulky pendant groups, which not only displayed mechanical properties higher than the strong and tough polymers, e.g., polycarbonate, but also excellent self‐repairing capability and recyclability as thermosets are developed. Owing to the stability of conjugation effect by aromatic benzene rings, the final polyimine networks are far more stable than the reported counterparts, exhibiting excellent hydrolysis resistance under both alkaline condition and most organic solvents. These polyimine materials with conjugation structure can be completely depolymerized into monomers recovery in an acidic aqueous solution at ambient temperature. Resulting from the bulky pendant units, this method allows the exchange reactions of conjugation polyimine vitrimer easily within minutes for self‐healing function. Moreover, the introduction of trifluoromethyl diphenoxybenzene backbones significantly increases tensile properties of polyimine materials. This work provides an effective strategy for fabricating high‐performance polymer materials with multiple functions.

## Graphical abstract

CO‐polyimine materials (PIMs) films are successfully synthesized by copolymerization and evaluated. Owing to the presence of *π*–*π* conjugation effects and steric effects as well as the high cross‐linking density of dynamic imine bonds, CO‐PIMs films as thermosets not only exhibit excellent mechanical strength and self‐healable behavior, water, and solvent resistance but also closed‐loop recycling.

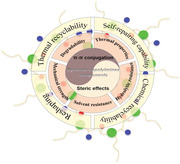

## 1 Introduction

Permanently cross‐linked thermosets with preferable thermo‐mechanical strength, dimensional stability, chemical resistance, and load carrying ability are widely produced and used in vehicles, aerospace composites, wind power, microelectronics, etc.^[^ ^1^ ^]^ However, reprocessing and recycling of thermosets‐polymeric materials is a major scientific challenge with the ever‐increasing production of plastic goods, and on account of not having melting nor dissolving characteristics.^[^ ^2^ ^]^ An increasing fraction of thermoplastics or thermoset plastics is recycled or incinerated to recover energy, but most ends up in landfills, littering cities or landscapes, and in the oceans, leading to severe environmental burden.^[^ ^3^ ^]^ Notably, in the future, flexible electronics will inevitably face the human interactive conditions (e.g., repeated bending, stretching, electrostatic discharge, and moisture) and unsatisfactory self‐healing properties (e.g., healing efficiency and rate) under the potentially harsh outdoor environments, which may lead to reduced reliability and a large amount of electronic waste.^[^ ^4^ ^]^ Under the guidance of circular economy and environmental sustainability, self‐healing and reprocessing of thermoset polymers based on cross‐linked network become highly desirable but intractable.^[^ ^5^ ^]^

Skin on living organisms share the ability to grow on their surface to achieve self‐healing and regenerative functions.^[^ ^6^ ^]^ Inspired by the ability of biological systems, considerable research efforts have been focused on recyclable and self‐healing artificial materials in recent years.^[^ ^7^ ^]^ The concept of Covalent Adaptable Networks (CANs), so‐called “Vitrimers” to sustainable development has gained significant interest as a viable solution, and is becoming an area of active research in self‐healable smart materials reported by Leibler and co‐workers since 2011.^[^ ^8^ ^]^ A series of dynamic covalent bonds and exchange such as esters,^[^ ^9^ ^]^ disulfides,^[^ ^10^ ^]^ boronic ester,^[^ ^11^ ^]^ vinylogous urethanes,^[^ ^12^ ^]^ Diels–Alder adducts,^[^ ^13^ ^]^ Schiff base,^[^ ^2b^ ^]^ vinylogous urethanes exchange,^[^ ^14^ ^]^ olefin metathesis,^[^ ^15^ ^]^ etc., have been incorporated into the CANs, which provide an interesting solution to the challenging recyclability of thermosets and elastomers.^[^ ^16^ ^]^ CANs combine excellent mechanical properties of thermosets with reprocessability, rehealability, and recyclability of thermoplastics, which can be reprocessed virtually and retain the quality of the parent polymers. In this regard, self‐healing polymers with CAN are expected to extend the lifetime, safety, and durability of polymeric materials, and have witnessed rapid progress in a short time window.

Among CANs, the imine bond‐containing matrix system, generated by “Sciff base reaction” reported by the German chemist Ugo Schiff in 1866,^[^ ^17^ ^]^ has gained a tremendous interest as it can be easily synthesized via the condensation reaction of commercially available aldehyde/ketone and amine, as well as for the potential to obtain these from bio‐based or carbon dioxide (CO_2_)‐based sources.^[^ ^18^ ^]^ Moreover, compared with some other dynamic covalent bonds, imine bond exchange reaction can occur in a mild condition (< 100 °C) with a high yield, and hydrolysis of imine linkages may be achieved in free amine solution or mild acidic solution. There are three types of reversible reactions including imine formation and hydrolysis, amine‐imine exchange, and imine‐imine exchange.^[^ ^16d^ ^]^ In addition, the feedstocks can be recoverable after these reversible reactions.^[^ ^19^ ^]^

Thermoset polyimines capable of mechanical, thermal, and chemical recycling are highly sought‐after to produce sustainable materials with a circular plastic economy.^[^ ^20^ ^]^ Recent developments in CANs‐based “smart” polyimine also showcase the potential for unprecedented applications beyond dynamic chemical structures, such as matrices for carbon fiber composites,^[^ ^19^, ^21^ ^]^ thermally moldable microcellular foams,^[^ ^22^ ^]^ adhesive on different substrates,^[^ ^23^ ^]^ aerogel for applications in thermal insulation and emulsion separation,^[^ ^24^ ^]^ anticorrosion coating,^[^ ^25^ ^]^ conductive composites,^[^ ^26^ ^]^ polyurethane based on antibacterial ability,^[^ ^27^ ^]^ and elastomer for stretchable electronic circuit^[^ ^28^ ^]^ owing to their repairability, weldability, closed‐loop recyclability, shape memory, and foamability. However, the conflict between high mechanical strength and self‐healing ability of thermosets remains a huge challenge to achieve a breakthrough in the application of electronics and energy devices. To date, much efforts has focused on exploring various properties of intrinsic polyimine materials, such as using novel crosslinker,^[^ ^29^ ^]^ constructing bio‐based polyimine vitrimer,^[^ ^30^ ^]^ introducing multifunctional curing agent,^[^ ^16^, ^31^ ^]^ imide‐imine hybrid,^[^ ^32^ ^]^ interpenetrating CANs (IPCANs),^[^ ^33^ ^]^ poly(imine‐carbonate)s (PImCs),^[^ ^34^ ^]^ supramolecular CANs (supra‐CANs),^[^ ^28c^ ^]^ etc. Despite some progress in high performance polyimine materials, it still remains a huge challenge to develop dynamic polyimine thermosets with mechanically robust properties.

Traditional polymers are often synthesized from different monomers for achieving synergy that is unavailable for single components.^[^ ^35^ ^]^ Meanwhile, the topological structures which delineate the bridges along the backbone of network components act in a critical role in dictating the properties of most thermosets as well.^[^ ^36^ ^]^ It is thus meaningful to impart controllable property and recoverability to the copolymerization of dynamic CANs.^[^ ^37^ ^]^ To the best of our knowledge, copolymerization with two diamines is a rarely explored method for designing recyclable polyimine materials. Sequence/properties‐controlled co‐polyimines (e.g., block copolymers, random copolymerization, etc.) remains a challenging issue. In addition, compared with classical polymer materials, fluoropolymers show remarkable differences in water absorptivity, thermal resistance, chemical stability, refractive index, permittivity, flammability, surface energy, and phase segregation. Fluoropolymers have quickly found commercially relevant applications, most notably in inert nonstick coatings,^[^ ^38^ ^]^ invisible plastic optics,^[^ ^39^ ^]^ and even as vessel grafts in biomedicine.^[^ ^40^ ^]^ Nowadays, perfluoropolyethers (PFPE) building blocks are widely used as reactive intermediates in the synthesis of a large range of materials for the creation of robust fluorinated networks, such as fouling‐release coating,^[^ ^41^ ^]^ fluorinated vitrimer elastomers,^[^ ^14^ ^]^ ion‐conducting PFPE‐based vitrimers,^[^ ^42^ ^]^ etc. Meanwhile, it has already been confirmed that the CANs materials with fluorinated groups exhibited accelerating effect of two exchange reactions and rapid self‐healing ability.^[^ ^43^ ^]^ However, the combination of polyimine CANs with fluorinated monomers has rarely been explored so far.

Enabled by the dynamic exchange of imine bonds, herein, we develop a polyimine vitrimer, also known as an associative CANs, with the ability of crosslinking and copolymerization. To examine structure–function relationships, we used two different diamines, namely 4‐phenyl ether‐1, 3‐diamine (2, 4‐ODA), and 1, 4‐bis(4‐amino‐2‐trifluoromethylphenoxy)benzene (6FAPB) to create a novel copolyimine network, which uncommonly integrates the rigidity of 2, 4‐ODA and flexibility of 6FAPB. To further explore the design and property space of co‐polyimines, the thermal stability, mechanical properties, chemical resistance, the recyclability, and self‐healing behaviors of the prepared resins were investigated systematically. Additionally, *π*‐conjugated structures endow the final polymers with the potential of electrical conductivity.^[^ ^44^ ^]^ Therefore, these excellent comprehensive properties would greatly enlarge the potential applications of the copolyimine resins in flexible conductors and electrodes.

## 2 Results and Discussion

### 2.1 Structure Characterization and Thermal Properties of CO‐PIMs

**Figure 1:**
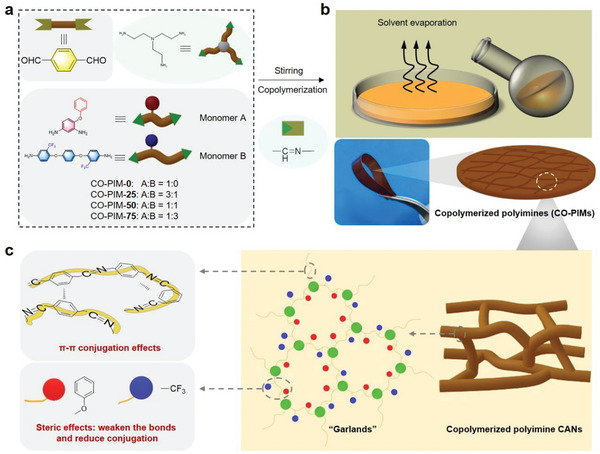
a) Illustrations of the synthesis of crosslinked CO‐PIMs with two types of diamines (2, 4‐ODA and 6FAPB) and TREN as the crosslinking unit. b) Schematic demonstration of the preparation process of the obtained CO‐PIMs films. c) Microcosmic component and architecture description of CO‐PIMs films.

Considering the future demand for high‐performance resins in industry, design, and development of mechanically robust resins without sacrificing the bond exchange capability is a challenging issue.^[^ ^21^, ^28^ ^]^ Aimed at this, a series of polyimine materials (CO‐PIMs) containing fluorinated blocks were designed and synthesized via copolymerization (Table S1, Supporting Information). After prepolymerization, the solution of polyimine oligomer was homogeneous (Figure S2, Supporting Information) due to these two diamine monomers with high bulky pendant units (Figure S1, Supporting Information). Resulting from the high reactivity between amino and aldehyde groups, the reaction systems form stable 3D CANs easily without a catalyst, and the microcosmic component and architecture of CO‐PIMs is like a series of “*Garlands*‐Like” (**Figure** **1**). In addition, the control experiment was conducted by using TA and 4, 4’‐oxydianiline (4, 4’‐ODA) to verify the function of 2, 4‐ODA with bulky pendant units (Figure S3, Supporting Information). The aldehyde group terminated polyimine oligomers using symmetrical 4, 4’‐ODA cannot be dissolved in NMP effectively, producing precipitate and gelation over time. To address this problem, 2, 4‐ODA was incorporated into the polyimine oligomers instead of 4, 4’‐ODA in our work.

**Figure 2:**
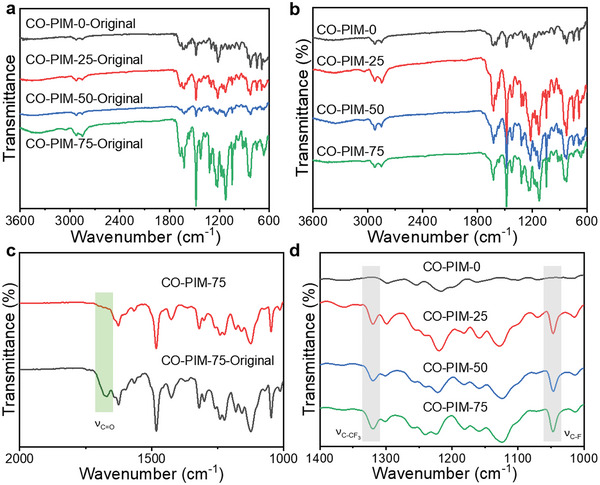
Attenuated total reflection Fourier transform infrared (ATR‐FTIR) spectra of CO‐PIMs. a) Original films without heating in a vacuum oven. b) Original films heated at 150 °C for 1 h under reduced pressure. c) Comparison on the CO‐PIM‐75‐Original and CO‐PIM‐75. d) An enlargement of the scope of wavenumber from 900 to 1200 cm^−1^.

The formation of our CO‐PIMs‐Original (copolymerized polyimine was heat‐treated only on 70 °C, Figure S4, Supporting Information) and CO‐PIMs was first proved by ATR‐FTIR. As shown in **Figure** **2a,b**, for all samples, the vibration peaks of the films at 2957 and 2872 cm^−1^ are attributed to the methyl and methylene stretching vibration in the TREN. The strong absorption peak around 1482 cm^−1^ is ascribed to the stretching vibration of benzene ring. The absorption peaks around 1220–1270 and 1000–1022 cm^−1^ are ascribed to the stretching vibration of C—O—C. In the case of CO‐PIM‐75‐Original and CO‐PIM‐75 (Figure 2c), a characteristic peak at around 1625 cm^−1^ appears and the signal at 1697 cm^−1^ disappears, which correspond to the stretch vibration of C=N double bond, and aldehyde groups of aromatic aldehydes, respectively. Above observations indicate high monomer conversion via chemical reaction between amines and aldehydes. Moreover, as shown in the enlarged image of Figure 2b (Figure 2d), the new peaks at around 1047 cm^−1^ of C—F moieties and 1320 cm^−1^ of C‐CF_3_ confirm that 6FAPB has been introduced into the main chain of polyimine materials.^[^ ^8d^ ^]^ As stated above, the similar curves of ATR‐FTIR suggest similar components and states of polymer chains in the materials under the unstrained condition, which is in accordance with our structural design. Well‐defined compositions of the polyimine copolymers in two different ratios of 2, 4‐ODA to 6FAPB were synthesized, demonstrating the synthetic versatility of such a copolymer system. Additionally, the swelling ratios of CO‐PIM‐0, CO‐PIM‐25, CO‐PIM‐50, and CO‐PIM‐75 were 0.93%, 0.61%, 0.86%, and 0.63%, respectively. The gel contents of CO‐PIM‐0, CO‐PIM‐25, CO‐PIM‐50, and CO‐PIM‐75 are 99.8%, 99.0%, 99.7%, and 99.8%, respectively, indicating the formation of the high density of the crosslinking network (Figure S5, Supporting Information). The cross‐linking features of CO‐PIMs networks are obtained from the typical kinetic theory of rubber elasticity (Figure S6, Supporting Information).^[^ ^22^ ^]^ Those high glass transition temperatures (*T*_g_s) in the temperature of 155–182 °C determined by the peak temperature in the tan *δ* curves (Figure S6b, Supporting Information) are mainly due to the aromatic benzene rings and *π*–*π* conjugation effects. CO‐PIMs exhibited high values of cross‐link density (*v*_e_) in the range of 833.3–1214.6 mol m^−3^ (Table S2, Supporting Information), and the lower *v*_e_ of CO‐PIM‐75 is probably due to the phenoxy chains with much larger free volume and rigid structures in 2, 4‐ODA.

**Figure 3:**
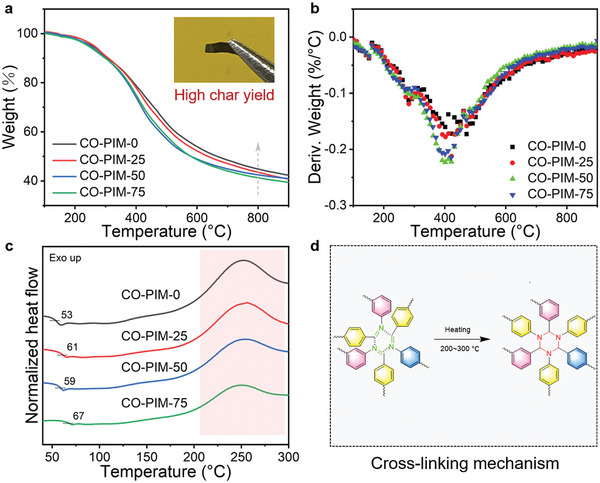
a) Nonisothermal TGA curves of CO‐PIMs. Inset: Images to show the char yield at 900 °C in N_2_. b) DTG curves of CO‐PIMs. c) Nonisothermal DSC curves of CO‐PIMs. d) Possible cross‐linking mechanism of aromatic Schiff structures.

The thermal properties of these four CO‐PIMs networks were investigated by thermogravimetic analysis (TGA) and differential scanning calorimetry (DSC) tests. TGA was applied to study the thermal stability of the polyimine resins in a N_2_ atmosphere. The TGA and derivative thermogravimetry (DTG) curves as a function of temperature are shown in **Figure** **3**. There is no much difference in the thermal stability between the prepared copolymerization resins (Figure 3a; and Table S2, Supporting Information), wherein 5% weight loss (*T*_d5_) all occur at the temperatures over 242 °C. The fastest degradation process in the range of 400–500 °C, is attributed to the breakage of chemical bonds in which the decomposition of ether bond occurs. Theoretically, the CO‐PIM‐0 containing more rigid benzene rings would increase the char yield at 800 °C (*R*_800_) of the resulting polyimine networks. As mentioned above, the *R*_800_ values of CO‐PIM‐0, CO‐PIM‐25, CO‐PIM‐50, and CO‐PIM‐75 are 44.8%, 43.3%, 42.2%, and 41.3%, respectively. The attractive thermal stability results from the nature of the benzene‐based network with imine bonds. As displayed by DSC results, *T*_g_s of CO‐PIMs are not high in the range of 53–67 °C. Overall, the similar thermal properties of the CO‐PIMs also suggests similar components and states of polymer chains in the materials. It should be noted that an obvious exothermic peak appears from 200 to 300 °C. This special exothermic peak for CO‐PIMs proves that the self‐crosslinking behavior caused by aromatic imine structures occurs in the temperature range in nitrogen, thereby facilitating the carbonization of materials (Figure 3d).^[^ ^45^ ^]^ The results can be further confirmed by DMA curves in the temperature range of 200–250 °C, which leads to an increasement in the modulus (Figure S6, Supporting Information). Meanwhile, the correlative enthalpy (Δ*H*) of CO‐PIMs decreases from 165.4 to 102.6 J g^−1^ with increasing the content of 6FAPB due to the longer molecular chains of 6FAPB compared with 2, 4‐ODA. The results were further verified by TG‐DSC measurement (Figure S7, Supporting Information). An obvious exothermic peak around 266 °C appears in the temperature range of the *T*_g_ and decomposition peaks. To the best of our knowledge, similar curing phenomenon of monolithic polyimine CANs by DSC and TG‐DSC has never been discussed in previous reports.

The topology freezing temperature (*T*_v_) is a key parameter of vitrimers, which corresponds to a high thermal expansion rate due to the exchange of dynamic bonds. When the temperature is below *T*_v_, the bond exchange reaction is so slow that the network structure is effectively frozen. Furthermore, it is critical for determining the relatively small useful processing window of vitrimers. As shown in Figure S8 (Supporting Information), *T*_v_ can be observed experimentally by dilatometry.^[^ ^42^ ^]^ The *T*_v_ values of CO‐PIM‐0, CO‐PIM‐25, CO‐PIM‐50, and CO‐PIM‐75 are found to be ≈223, ≈223, ≈203, and ≈168 °C, respectively. The different *T*_v_ values provide the guidance for reprocessing temperatures. As expected, our network with its degrees of freedom constrained by *π*–*π* conjugation effects and robust segments in 2, 4‐ODA shows a lower thermal expansion coefficient than the counterpart system.

### 2.2 Mechanical Properties of the CO‐PIMs

**Figure 4:**
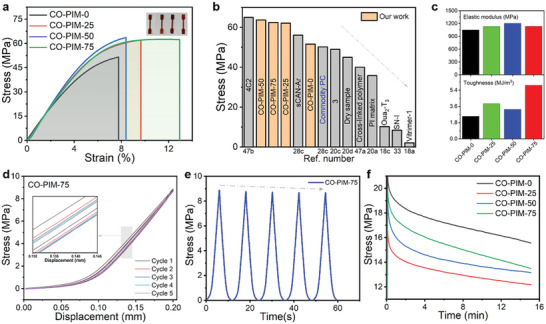
Mechanical properties of CO‐FPIVs. a) Stress–strain curves of CO‐PIMs were recorded with a deformation rate of 2 mm min^−1^. b) Comparison of the stress of CO‐PIMs and a range of other TREN‐based polyimines, including commodity PC product. Inset: specimen name in the reported references. c) Elastic modulus and toughness of CO‐PIMs calculated from their stress–strain curves. d) Stress‐displacement behavior in cyclic tensile test curves of CO‐PIM‐75 recorded with constant strains of 0.2 mm. e) Stress‐time in cyclic tensile test curves of CO‐PIM‐75 recorded with constant strain of 0.2 mm. f) Typical stress relaxation curves of CO‐PIMs recorded with a function of time during the relaxation process at a prestretched strain of 0.2 mm.

In the preceding sections, our CO‐PIMs have been synthesized successfully, and the introduction of 6FAPB improves the *T*_g_ values. To date, the advantages of copolymerization in regulating mechanical properties of polymer have been confirmed. Herein, we investigate whether the similar mechanical property is also presented in the CANs like polymer materials. The differences in mechanical properties for these four kinds of networks were investigated by tensile tests to disclose the features of the copolymerzation crosslinks. The uniaxial tensile testing at a strain rate of 2 mm min^−1^ was used to evaluate the mechanical properties of the as‐prepared resins. The representative stress–strain curves of CO‐PIM with different 6FAPB content and neat CO‐PIM‐0 are shown in **Figure** **4**a, comprehensive comparison of the stress between the reported TREN‐based polyimines and commodity polycarbonate (PC) is shown in Figure 4b, and the corresponding elastic modulus and toughness are shown in Figure 4c. It could be found that the CO‐PIM‐0 specimen without flexible 6FAPB is slightly stiff and relatively brittle due to the rigid and shorter chain length of 2, 4‐ODA. The tensile strength of the CO‐PIM‐0 specimen is measured to be 51.6 MPa, with an elastic modulus of 1049.8 MPa, and an elongation at break of 7.8%. Thus, a flexible 6FAPB was introduced to improve the mechanical properties by forming a cross‐linked structure. After copolymerization with 6FAPB, the CO‐PIMs are more ductile and tougher. When the molar ratio of 2, 4‐ODA to 6FAPB is increased from 0:1 to 3:1, the strain at the break has an opposite trend. Not only the elongation at break increases, but also the tensile strength and elastic modulus increase significantly. Impressively, the tensile strength and elongation at break of CO‐PIM‐50 specimen reach ≈62.9 MPa and *≈*8.4%, which are 21.9% and 7.7% higher than those of the CO‐PIM‐0 specimen, respectively. As for CO‐PIM‐75, the tensile strength (62.5 MPa) and elongation at break (12.9%) are 21.1% and 65.4% higher than those of the CO‐PIM‐0 specimen, respectively. It should be noted that in terms of toughness, CO‐PIM‐75 (5.94 MJ m^−3^) show 2.37 times increase than CO‐PIM‐25 (2.50 MJ m^−3^). Thus, the enhanced mechanical properties of the resins can be facilely regulated by changing the content of 6FAPB. This phenomenon is mainly attributed to the introduction of more flexible and longer chain of 6FAPB, resulting in increased the flexible distance of cross‐linking sites. In comparison, the tensile strength of CO‐PIMs with high aromatic composition is higher than the commodity PC^[^ ^28c^ ^]^ which is a characteristic high‐performance engineering plastic with outstanding strength and stiffness. These results suggest that CO‐PIM possessed the advantages of 2, 4‐ODA and 6FAPB in tensile properties simultaneously, thereby exhibiting better mechanical performance.

Subsequently, the antifatigue property of the CO‐PIM‐75 network was explored by performing five cyclic stress–strain tests at a constant strain of 0.2 mm without an interval (Figure 4d). Overall, CO‐PIM‐75 displays good recovery properties as shown by the cyclic tensile tests with different rest intervals ranging from 0 to 0.2 mm. In detail, on one hand, the maximum stress in the subsequent cyclic stretching gradually decreases slightly after the 1st cyclic stretching (Figure 4e), which is ascribed to the facture of some reversible imine bonds during the stretching process. On the other hand, after the initial loading/unloading process, every cycle without relaxing exhibits a reduced hysteresis loop because the broken dynamic covalent bonds interactions cannot rebond timely.^[^ ^43c^ ^]^ The stress relaxation curves of CO‐PIMs in Figure 4f as a function of applied tensile time is clearly divided into two sections. Initially, the stress increases exponentially within several seconds until the strain reaches 0.2 mm, and then the strain keeps constant. The stress decreases gradually with the time and tends to reach equilibrium due to the network confinement after 15 min.

### 2.3 Insights into Hydrolytic Resistance and Solvent Resistance

**Figure 5:**
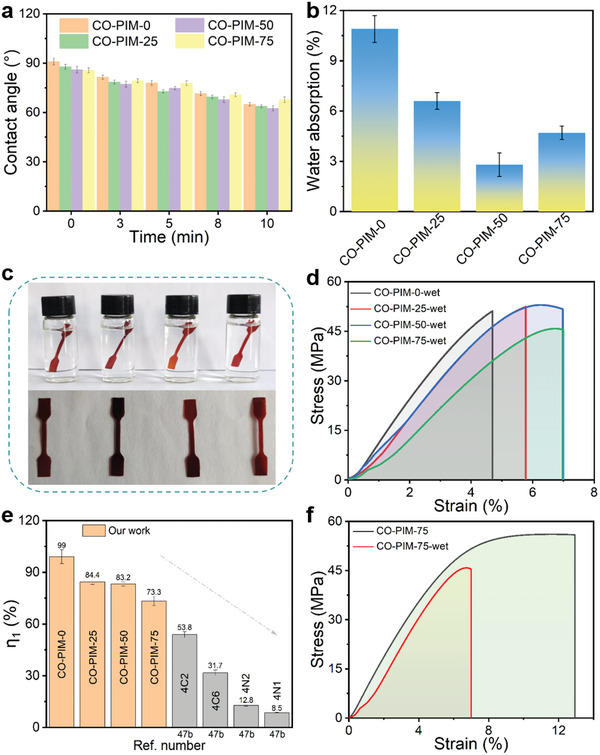
Hydrolysis resistance of CO‐PIMs. a) Water contact angles of CO‐PIMs with different time. b) The water absorption of CO‐PIMs soaked in deionized water after 1 week. c) Photographs to show the sheets of CO‐PIMs soaked in deionized water after 24 h for tensile tests. d) Stress–strain curves of the wet CO‐PIMs samples. e) Comparison of the strength retaining efficiency (*η*_1_) of CO‐PIMs‐wet and a range of other polyimines reported by Zhang and co‐workers.^[^ ^47b^ ^]^ f) Comparing the representative tensile stress‐strain curves of the CO‐PIM‐75 and wet CO‐PIM‐75 samples.

The imine bond is a covalent bond with strong polarity and good hydrophilicity.^[^ ^46^ ^]^ Thus, in addition to mechanical properties, hydrolytic and solvent resistance are also important for polyimine thermosets. To this end, we first studied the hydrolytic resistance of CO‐PIM through qualitative and quantitative tests. **Figure** **5**a shows that the water contact angles of CO‐PIMs films are close to 90° upon contacting the surface of CO‐PIMs films, suggesting that these films are hydrophilic. Afterward, the water contact angle is reduced with increasing time, the water contact angles of CO‐PIM‐0, CO‐PIM‐25, CO‐PIM‐50, and CO‐PIM‐75 decrease by 28.57%, 27.33%, 27.33%, and 20.89% in 10 min, respectively. The results are due to hydrolysis of the partial Schiff base and plasticization by the absorbed water. Furthermore, we found the CO‐PIMs cannot be dissolved in deionized water at ambient temperature even for 1 week, exhibiting excellent hydrolysis resistance. In detail, the water absorption rate of CO‐PIM‐50 shows a decrease from (10.9 ± 0.8)% to (2.8 ± 0.7)% as compared to the CO‐PIM‐0 vitrimers (Figure 5b). The CO‐PIMs with more 6FAPB displays superior hydrolytic resistance after immersed in water, which could be assigned to the effect of conjugation structure and —CF_3_ units.^[^ ^43c^ ^]^

To deeply figure out the hydrolytic behaviors, we also employed quantitative measurements to evaluate the influences of hydrolysis on the mechanical performance of CO‐PIMs samples. The dumbbell‐shaped films were used to prepare the test samples, which were treated at ambient temperature by soaking into deionized water environments for 24 h. Figure 5c also presents the results for the samples treated with hydrolytic adsorption. The color of the samples does not change and still reddish brown after immersing in deionized water. To further observe the change of the chemical structure of CO‐PIMs by immersing in deionized water (CO‐PIMs‐wet), ATR‐FTIR spectroscopy measurements were conducted. It is found that the ATR‐FTIR spectra of CO‐PIMs‐wet are similar (Figure S9a, Supporting Information), the new weak peak at 1697 cm^−1^ may belong to the aldehyde groups of aromatic aldehydes unit of partly hydrolyzed aldehyde groups (Figure S9b,S9c, Supporting Information).

Impressively, the stress–strain curves and corresponding summary data show that the mechanical properties of the hydrolytic materials can be well maintained compared with those of the pristine material. Compared with the previous report by Zhang and co‐workers (lose > 90% original tensile strength),^[^ ^47^ ^]^ it is noteworthy that the mechanical properties of our samples always maintain at a high level in a wet state (>73% original tensile strength) (Figure 5e,f). Moreover, the wet samples with high content of —CF_3_ exhibit low retention rate of mechanical properties, demonstrating the accelerating effect of two exchange reactions by the introduction of 6FAPB under water environment.^[^ ^43^ ^]^ Owing to the conjugation effect, it is likely that the equilibrium condition involves hydrolysis of only a small proportion of imine linkages, revealing high feasibility of practical application, especially in high‐humidity atmosphere.

The polar ether bonds and bulkily pendant group in the structure of diamine monomers in this work may enhance the swelling ability of the resulting polymer in polar solvent and increase the possibility of chemical degradation. However, the covalently cross‐linked network of the prepared CO‐PIM‐75 samples can be further confirmed by the fact that it is insoluble in most organic solvents. The rectangular samples (≈10–20 mg) were added into 2 g of methanol (MeOH), ethyl alcohol (EtOH), tetrahydrofuran (THF), dichloromethane (EtOAc), ethyl acetate, dimethylformamide (DMF), N‐methylpyrrolidone (NMP), respectively (Figure S10, Supporting Information). After 1‐week immersion in these organic solvents, the mass change ratios of CO‐PIM‐75 before and after soaking in different organic solvents under the ambient environment are shown in Table S4 (Supporting Information). The shape of rectangular samples retains and the color of solvents does not change much in MeOH, EtOH, THF, EtOAc, and NMP, which are due to the conjugation between molecular chains. In contrast, some of the reported Schiff base vitrimers can be easily dissolved in organic solvent systems at ambient temperature. Therefore, resulting from the high chemical stability of the as‐prepared materials in this work, the CO‐PIM‐75 is relatively stable in various organic solvents.

Schiff base vitrimer is usually regarded as an acid‐sensitive material since the imine condensation and hydrolysis reactions are reversible. To demonstrate the durability of the CO‐PIM‐75 system, the acid and alkaline resistance of the Schiff base materials was also evaluated. After 1‐week immersion in an aqueous solution of 0.1 m NaOH and 0.1 m HCl, the shape of rectangular samples all retains (Figure S11, Supporting Information). Resulting from the conjugation effects between an imine and aromatic rings, the stability of an imine prepared from an aromatic amine and an aldehyde is superior to that of aliphatic companions.^[^ ^48^ ^]^ The color of NaOH solution does not change much, but the color of HCl solvent becomes light yellow, which TREN HCl dissolved in the aqueous mixture.^[^ ^18d^ ^]^ Notably, after depolymerization in 1 week, many microvoids and microcracks on the surface of rectangular samples in 1 m HCl are observed (Figure S11d,S11e, Supporting Information). Under acidic conditions, CANs containing imine bonds can be degraded to oligomers containing amine and aldehyde for recovery,^[^ ^5^, ^29^, ^43^ ^]^ and the degradation mechanism of our CO‐PIMs with conjugated structures immersing in acid solution is shown in Figure S12 (Supporting Information). In a word, CO‐PIM‐75 is stable in neutral or alkaline conditions but hydrolyzes in weak acidic conditions.

### 2.4 Reshaping, Self‐Healing, and Reprocessability of CO‐PIMs

According to the malleability of the polyimine material, the corresponding polyimine strip can be reshaped under heat stimulus which induce the exchange of imine bonds at the molecular level, thereby reducing the viscosity to achieve reshaping. The flat CO‐PIM‐75 strip can be reshaped into a twisted strip and back into its original flat shape in only a few minutes of heating in 80 °C (Figure S13, Supporting Information). In this case, it exhibits great potential to realize more complex reprocessing procedures obtaining desired shapes for our CO‐PIM‐75 with malleability.^[^ ^49^ ^]^

**Figure 6:**
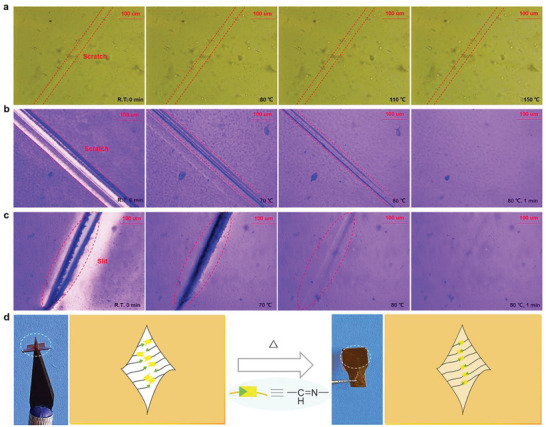
Schematic of the proposed self‐healing process of polyimine copolymers which show the healing behavior of the cracked films by reformation of imine bonding. Optical microscope images to show self‐healing process of a) Shallow scratch on the surface of CO‐PIM‐75 film. b) Shallow scratch on the surface of CO‐PIM‐75‐Origin film, and c) Slit on the surface of CO‐PIM‐75‐Origin film. d) Images and mechanism of the self‐healing process of CO‐PIMs.

To date, the advantages of CO‐PIM in ensuring mechanical properties, water and chemical resistance of materials have been confirmed. Then we investigate whether the dynamic property is also presented in the CO‐PIM crosslinks like that of CAN materials. As is well known, biological systems can heal themselves autonomously and regenerate their structures and original set of properties after suffering from external mechanical damage. Inspired by the ability of biosystems to heal wounds autonomously, the self‐healing performance of CO‐PIM‐75 as a representative was evaluated. The self‐healing process of polyimine copolymers were investigated by polarizing microscope (POM), as recorded in **Figure** **6**. The shallow scratch on the CO‐PIM‐75 film could self‐heal after 1 h of healing from ambient temperature to 150 °C. It should be noted that the self‐healing property of CO‐PIM‐75 film was not obviously examined with heating when the surface with deep scratch (Figure 6a). Subsequently, we also explored the self‐healing process of CO‐PIM‐75‐Origin further. The deep and wide scratch (≈120 µm) on the surface of CO‐PIM‐75‐Origin film that would be clearly observed by POM almost completely disappear within about 1 min upon heating from ambient temperature to 80 °C (see Figure 6b; and Movie S1, Supporting Information). Moreover, we made a slit (≈60 µm) in the middle of CO‐PIM‐75‐Origin film to explore the self‐healing capability of the broken film. To our delight, the dimension of slit became increasingly smaller triggered by thermal stimuli, resulting in self‐healing completely within only several minutes (see Figure 6c,d; and Movie S2, Supporting Information). The self‐healing process of this synthetic polymer is similar to natural healing of wounds and cuts in the biological organisms.^[^ ^21^, ^50^ ^]^ This remarkable phenomenon is in good agreement with the preliminary molecular study that highlighted the strong activation of imine bonds exchange induced by fluorine atoms,^[^ ^43^, ^51^ ^]^ and the size of the bulky groups may weaken the imine bonds (Figure S14, Supporting Information).^[^ ^16d^ ^]^ On the molecular level, the imine exchange occurs two timescales, first to imine exchange in close proximity, and second then to imine exchange in the distant proximity as a result of diffusion through the CANs on a longer timeframe. The introduction of trifluoromethyl diphenoxybenzene dackbones with polar groups and more flexible chains is helpful to reduce the charge transfer complex (CTC) and stiffness within the aromatic molecules. This leads to the relaxation in the network topology and enhanced “slithering” process to achieve the excellent self‐healing for aromatic polyimines (Figure S15, Supporting Information).^[^ ^52^ ^]^

**Figure 7:**
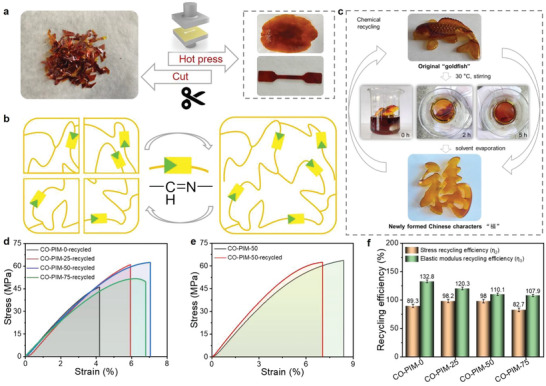
a) Diagram showing the thermal recycling experiment of CO‐PIM‐75. b) The mechanism of thermal recycling of the CO‐PIMs. c) Digital photographs of the closed‐loop recyclability of CO‐PIM‐75‐Original plastic with gold‐fish shape. d) Stress–strain curves of the thermal recycled CO‐PIMs samples. e) Comparing the representative tensile stress–strain curves of the CO‐PIM‐50 and the thermally recycled CO‐PIM‐50 samples. f) The bar chart of stress recycling efficiency (*η*_2_) and elastic modulus recycling efficiency (*η*_3_) of CO‐PIMs.

Subsequently, physical recycling of the polyimine copolymers through hot press was studied. The original thermoset films were cut into pieces and then hot‐pressed at 150 °C for 10 min under 10 MPa, after which a remolded specimen can be successfully obtained (**Figure** **7**a,b, Supporting Information).^[^ ^5c^ ^]^ As shown in the tensile results in Figure 7d,e, there is a slight decrease in stress (Figure 7f), elongation at break and toughness with the reprocessing (Figure S17a,b, Supporting Information), but a slight increase in elastic modulus (Figure 7f). During the process of hot press, imine bonds show two more reversible exchange reactions simultaneously: imine metathesis occurring between two imines, and transamination reaction requiring free amine groups (Figure S18, Supporting Information).^[^ ^53^ ^]^ This slight alteration can be explained by some side reactions (e.g., self‐crosslinking^[^ ^45a^ ^]^ or hyper‐cross‐linked polyaminal^[^ ^54^ ^]^) with hot pressing, leading to an increase of crosslinking density and increased brittleness of the materials. Different from self‐healing, reprocessability has a higher requirement for the dynamicity of crosslinks.^[^ ^5^, ^18^ ^]^ To our delight, the mechanical stiffness and strength of the reprocessed CO‐PIMs are also superior to those of the widely used commercial PC. The results in this study prove that the inherent trade‐off between robustness and dynamicity can be effectively circumvented though copolymerization with two diamines. Overall, the unique structural and dynamic characteristics of the CO‐PIMs endow the resultant CO‐PIMs with excellent reprocessability.

Finally, we explored the closed‐loop recyclability of representative CO‐PIM‐75‐Original by adding an excess of free amine solution (Figure S19, Supporting Information). As indicated in Figure 7c, under stirring at 30 °C for 5 h, the “goldfish” shape of CO‐PIM‐75‐Original with crosslinking network is depolymerized into soluble monomers and oligomers,^[^ ^24a^ ^]^ and completely dissolves in the recycling solution. Impressively, the new shape “
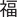
” of Chinese character can be regenerated by the chemical recycled CO‐PIM‐75‐Original. The corresponding recycling process and mechanism of closed‐loop recyclability are shown in Figure S20 (Supporting Information).

## 3 Conclusion

In summary, we reported a copolyimine network crosslinked by amines and aldehydes without any catalyst, which has uncommonly integrated advantages of 2,4‐ODA and 6FAPB. All CO‐PIMs showed high thermal stability with maximum decomposition temperatures of ≈400 °C and *R*_800_ > 41%. By tuning the equivalent ratio of 2,4‐ODA/6FAPB in the copolymerization system, the tensile properties of CO‐PIMs could be improved effectively, which is comparable to strong and tough plastic PC. Remarkably, the CO‐PIM‐50 exhibit a high tensile strength and strain of 63.7 MPa and 8.4%, respectively. The crosslinked polyimine based on this copolymerization system exhibited comparable hydrolysis and solvent resistance to many other stiff vitrimers and thermosets. Compared with the original samples, there was only negligible decrease in tensile properties for the recycled polyimine thermosets with hot‐pressing and the wet polyimine thermosets immersing in deionized water. In addition, the CO‐PIMs also possesses dynamic properties like those of polyimine materials with good self‐healing and reprocessability. Overall, these materials with unique structure exhibit unique performance and function. This work unveils a general approach to design polyimine networks, and this environmentally‐friendly plastic with excellent integrated properties can provide a new perspective on designing smart CANs at the molecular level.

## 4 Experimental Section

All detailed experimental section and methods that support the findings of this study are provided in the Supporting Information.

## Conflict of Interest

The authors declare no conflict of interest.

## Supporting information

Supporting Information

Supporting Information

Supporting Information

## Data Availability

The data that support the findings of this study are available from the corresponding author upon reasonable request.
