# Supplementary material for: Mechanically Robust, Recyclable, and Self‐Healing Polyimine Networks
Source: Adv Sci (Weinh). 2023 Apr 23;10(19):2300958. doi: 10.1002/advs.202300958 (PMC10323645; doi:10.1002/advs.202300958)
Supplement: Supplementary file 1 — Supporting Information [file ADVS-10-2300958-s002.pdf]

ADVANCED SCIENCE
Open Access

Supporting Information

for *Adv. Sci.*, DOI 10.1002/advs.202300958

Mechanically Robust, Recyclable, and Self-Healing Polyimine Networks

Ping Yu, Haiyue Wang, Ting Li, Guimei Wang, Zichen Jia, Xinyu Dong, Yang Xu, Qilin Ma, Dongen Zhang, Hongliang Ding and Bin Yu**

Supporting Information

Mechanically Robust, Recyclable and Self-Healing Polyimine Networks

Ping Yu, Haiyue Wang, Ting Li, Guimei Wang, Zichen Jia, Xinyu Dong, Yang Xu, Qilin Ma, Dongen Zhang, Hongliang Ding, and Bin Yu**

Table of Contents

1. Materials and General Methods..3

2. Synthesis and Characterization of Model Compounds..7

3. Supplementary Schemes & Figures..9

4. References..18

Section 1. Materials and General Methods

All reagents were commercially available and used as supplied without further purification. The following chemicals and reagents employed were purchased commercially as follows. Terephthalaldehyde (TA, 98%) and tris(2-aminoethyl)amine (TREN, 98%) were purchased from Aladdin Reagent Co., Ltd, China. 4-phenyl-ether-1,3-diamine (2, 4-ODA, 99%) were purchased from Suzhou Kareman New Materials Co., Ltd, China. 1, 4-Bis(4-amino-2-trifluoromethylphenoxy)benzene (6FAPB, 99%) were purchased from Bohai Chemical Co., Ltd., China. 1-Methyl-2-pyrrolidinone (NMP, 99.5%) were purchased from Beijing Innochem Science & Technology Co., Ltd, China. Methanol (MeOH), ethanol (EtOH), tetrahydrofuran (THF), dichloromethane (DCM), ethyl acetate (EtOAc), and dimethylformamide (DMF) were purchased from Tianjin Yongda chemical reagent Co., Ltd, China. All reactions were performed under ambient laboratory conditions, and no precautions are taken to exclude atmospheric moisture unless otherwise specified.

Fourier Transform Infrared Spectroscopy

Fourier transform infrared spectroscopy was conducted using a Nicolet iS10 Fourier-transform infrared spectrometer with an attenuated total reflection (ATR) attachment, and the scan ranges from 600 to 4000 cm^{-1} with 32 scans at a spectral resolution of 4.0 cm^{-1}.

Thermogravimetric Analysis

The thermogravimetric analysis (TGA) and cross-linking behaviors of CO-PIMs were performed on using a Netzsch simultaneous TG-DSC (449 F3) under N_2 flow (30 mL/min).[S1] Samples (5~10 mg) were heated from 50 to 900 °C with a rate of 20 °C/min. To observe the morphology of CO-PIM-75 samples after TGA-DSC tests, the aluminium crucible after tests was opened.

Differential Scanning Calorimetry

Differential scanning calorimetry (DSC) measurements were carried using on a NETZSCH DSC 214 instrument. Approximately 8 mg of samples was placed in a pierced aluminum pan, and the test was performed from 30 to 300 °C under a N_2 flow with a heating rate of 10 °C/min. Glass transition temperature (T_g) was obtained from the change in slope during the first heating curve.

Degradation Experiment

Around 80 mg of CO-PIM-75 vitrimer was immersd into a solution of 0.1 M acid (HCl+H_2O) and 0.1 M alkali (NaOH+H_2O) in a glass vial, respectively. The degradation behavior of samples was observed at ambient temperature in one week.[S2]

Swelling Ratio and Gelation Content Experiment

The dry CO-PIMs (~45 mg) were weighed (m_0) and soaked into glass vials with EtOH (~2.5 g) under ambient temperature. Then the samples were taken out after 72 h followed by wiping the residual solvent on the surface, and the mass was weighed (m_s). Finally, the samples were dried in a vacuum oven at 70 °C until the weight (m_d) remained unchanged. The swelling ratio (*SR*) and gelation content (*G*) was calculated as following equations:[S3]

$$SR = \frac{\mathrm{m_s} - \mathrm{m_0}}{\mathrm{m_0}} \times 100\% \quad (1)$$

$$G = \frac{\mathrm{m_d}}{\mathrm{m_0}} \times 100\% \quad (2)$$

Dynamic Mechanical Analysis

Dynamic mechanical analysis (DMA) was conducted on a DMA Q800 apparatus (TA Instruments). Tests were done in a tension film mode with a fixed frequency of 1 Hz, a strain amplitude of 20 μm, a preload force of 0.01 N, and 125% force track. Samples were scanned from -20 to 250 °C at a rate of 5 °C/min. The cross-linking density (v_e) was calculated by the

equation of $E^{'} = 3v_{\mathrm{e}}RT$, where $E^{'}$ is the modulus of rubbery plateau by DMA, R is the ideal gas contant (8.314 J mol^{-1} K^{-1}), and T refers to the absolute temperature with (T_g + 40) °C.[S4]

Dilatometry Experiments

Dilatometry and the value of topology freezing transition temperature (T_v) were determined using a DMA Q800 (TA Instruments) apparatus in a tension film geometry. Loaded with a weak elongational force of 11 kPa, the rectangular specimen with a dimension of ~10×4×0.2 mm^3 was scanned from -20 to 300 °C with a heating rate of 5 °C/min.[S5]

Tensile Tests of Dry Samples

The mechanical measurements were performed using a tensile tester (Zwick/Roell Z2005 universal testing machine with a 200 N sensor) at 25 °C with the relative humidity of 46%. Unless otherwise noted, all the test samples were dumbbell shaped (effective length: 12 mm, width: 2 mm, and measured thickness around 0.3 mm). The tensile curves were measured at a constant rate of 2 mm/min and at least five specimens were tested for each sample. The elastic modulus of the films was determined by calculating the slope of the initial position of monotonic tensile stress-strain curves. Stress relaxation tests for the CO-PIMs were performed with a predefined 0.2 mm strain at a deformation rate of 2 mm/min, and then the strain was kept constant at ambient temperature. The cyclic tensile test of CO-PIM-75 was repeated five times by stretching the films to 0.2 mm at a rate of 2 mm/min and then unloading it.

The tensile toughness (τ), a parameter that characterizes the work required to fracture the sample per unit, was calculated by the area surrounded by stress (σ)-strain (ε) curves, using the following equation:[S6]

$$\tau=\sum_{i=0}^{i=\varepsilon_b} \sigma\varepsilon_i \qquad (3)$$

where ε_i is the tensile stress, ε_b is the elongation at break.

The Contact Angle Measurement

The hydrophobicity of the films was studied on a contact angle analyzer (DSA100, Germany). The change of contact angle measurement with time (0 min, 3min, 5min, 10min) was recorded.

Tensile Tests of the CO-PIMs Films with Moisture

Pristine polyimine films with dumbbell shaped (effective length: 12 mm, width: 2 mm, and measured thickness around 0.3 mm) were prepared in the manner described above. At ambient temperature, the pre-tared samples were immersed into sample bottles filled with deionized water. After 24h, upon removal of the water from the surface of samples within 1 min, the wet tensile samples were measured at 25 °C with the relative humidity of 46% immediately.[S7] The

strength retaining efficiency (η_1) of wet sample was calculated by the ratio of tensile strength of the recycled sample (σ_w) to that of the original sample (σ_O).[S8]

$$\eta_1 = \frac{\sigma_w}{\sigma_O} \times 100\% \qquad (4)$$

Solubility and Chemical Resistance Tests

The rectangular CO-PIM-75 samples (10~20 mg) were separately immersed in different solvents (including MeOH, EtOH, THF, DCM, EtOAc, DMF and NMP) at ambient temperature for 7 days. Afterward, the solid and liquid phase were separated. Upon removal of the solvent from the surface of samples, the remaining mass was determined. The mass change rate (D%) was calculated according to the following equation: $D\% = (1 - W_2/W_1) \times 100\%$, where W_1 is the initial mass of the CO-PIMs before soaking, and W_2 is the mass of the CO-PIMs after soaking for 7 days in the ambient environment.

Reshaping of Thermoset

A simple experiment was conducted by heating at 80 °C and cooling to fix the deformed shape of strip.

Self-healing Tests

The images of optical microscopy (CPV-601C) were used for assessing the self-healing property of the synthesized co-polyimine samples against damage. First, a small visible scratch on the surface of the CO-PIM-75 films, then the damaged films covered with glass slides were placed on a heating table from 30 to 150 °C. Second, to assess the self-healing ability of the films containing the reversible imine bonds under varying conditions of damage. Tool of surgeon's knife was used for generating the deep scratch and long crack on the surface of CO-PIM-75-original without vacuum heating, respectively. Finally, the levels of damage and restoration were also examined using optical microscopy on a heating table from 30 to 80 °C.

Thermal Recyclability Experiments

A plate vulcanizer (QLB-50T) was used as the reprocessing recycle. The virgin films were cut into small pieces and placed between two steel sheets covered with two polyimide (PI) films. The reprocessing procedure was hot pressed with 10 MPa of pressure at 150 °C for 10 min to obtain a newly testable film.[S9] Afterwards, the tensile measurements of thermoset CO-PIMs with one round of reprocessing recycling were performed on a tensile tester (Zwick/Roell Z2005 universal testing machine with a 200 N sensor). The strength recycling efficiency (η_2) was calculated by the ratio of tensile strength of the recycled sample (σ_R) to that of the original sample (σ_O).[S8] The elastic modulus recycling efficiency (η_3) was calculated by the ratio of elastic modulus of the recycled sample (E_R) to that of the original sample (E_O). The toughness

recycling efficiency (η4) was calculated by the ratio of toughness of the recycled sample (τ_R) to that of the original sample (τ_O).

$$\eta_2 = \frac{\sigma_R}{\sigma_O} \times 100\% \qquad (5)$$

$$\eta_3 = \frac{E_R}{E_O} \times 100\% \qquad (6)$$

$$\eta_4 = \frac{\tau_R}{\tau_O} \times 100\% \qquad (7)$$

Chemical Recyclability Experiments

For chemical recyclability experiments, the recycling solution was composed of 15.0 mg of 2, 4-ODA, 96.3 mg of 6FAPB and 36 g of NMP (Figure S14). Then the goldfish-like CO-PIM-75 plastic was added in the recycling solution, and the mixture was stirred at 30 °C until complete dissolution.[S10] Then, 80.55 mg of TA was added into the solution to consume the free amine groups. After stirring for 0.5 h, 29.3 mg of TREN was added into the solution for 30 s resulting in the formation of the chemical recycled polyimine solution. Finally, the chemical recycled polyimine solution was poured into silicone abrasives with Chinese character "福" shape, and solvent evaporation with drying oven of 70 °C for 48 h.

Section 2. Synthesis and Characterization of Model Compounds

(a) Typical Procedure for the Preparation of CO-PIMs

It was synthesized via condensation polymerization, in which a CAN based on two diamine monomers and TREN. The general scheme adopted for the synthesis of the polyimine copolymer is shown in Scheme 1. Terephthalaldehyde and 1-Methyl-2-pyrrolidinone in a sample bottle was stirred at ambient temperature for dissolving completely. To determine the effect of copolymerization on properties, a certain amount of 2, 4-ODA and 6FAPB were added under 2 h of stirring until they were dispersed and reacted completely. Then, we obtained a black-to-orange and homogeneous solution with the increased content of 6FAPB. TREN and the residual NMP with solid content of 10 wt% were added to the vial containing the prepolymer under accelerating stirring for 30 s. The solution was then poured into glass disc quickly. After solvent evaporation at 70 °C for 2 days. A yellow thin film was obtained after peeling it off from the glass disc upon cooling to ambient temperature. According to our latest work,[S11] post-curing temperature is an important parameter in the field of conjugation polyimine materials. Thus, the target films in this work were cut into a dumbbell-shaped spline, and heated for 1 h under reduced pressure at 150 °C for subsequent testing. The molar ratio of CO-PIM-0, CO-

PIM-25, CO-PIM-50, and CO-PIM-75 were listed in Table S1. The films with different mass ratios of 2, 4-ODA to 6FAPB were denoted as CO-PIM-0, CO-PIM-25, CO-PIM-50 and CO-PIM-75, respectively. Therefore, all the yields of the homogeneous and red brown films for CO-PIM-0, CO-PIM-25, CO-PIM-50 and CO-PIM-75 were over 95%. Meanwhile, the thickness of the CO-PIMs films can be adjusted by controlling the concentration of copolyimine networks in the NMP solvent.[S12]

(b) Comparative Experiment on the Preparation of Polyimine Oligomer by Using 4, 4'-ODA

The aldehyde groups terminated polyimine oligomer by using TA and symmetrical 4, 4'-ODA was prepared according to the method of CO-PIM-0 material, and the dosage of TA and 4, 4'-ODA monomers was the same as the TA and 2, 4-ODA monomers of CO-PIM-0, respectively.

Table S1. Constitutions of various CO-PIMs vitrimers with copolymerization.

Compounds	TA (equiv)	2, 4-ODA (equiv)	6FAPB (equiv)	TREN (equiv)
**CO-PIM-0**	12	6	0	4
**CO-PIM-25**	12	4.5	1.5	4
**CO-PIM-50**	12	3	3	4
**CO-PIM-75**	12	1.5	4.5	4

Section 3. Supplementary Schemes & Figures

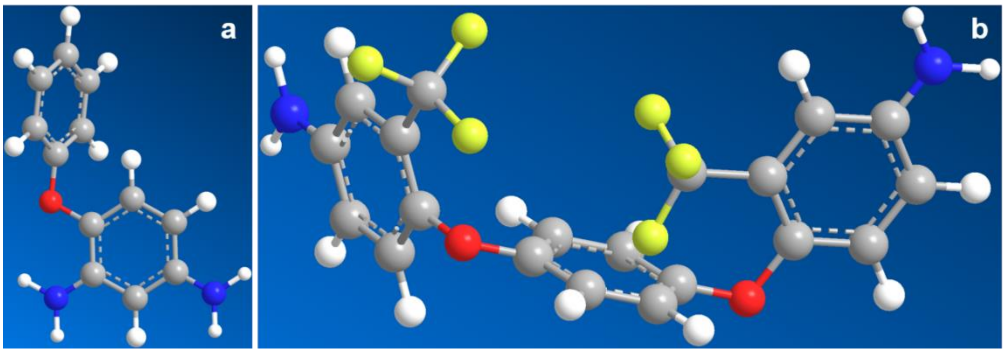

Figure S1. Molecular structure model of diamines: a) 2, 4-ODA and b) 6FAPB.

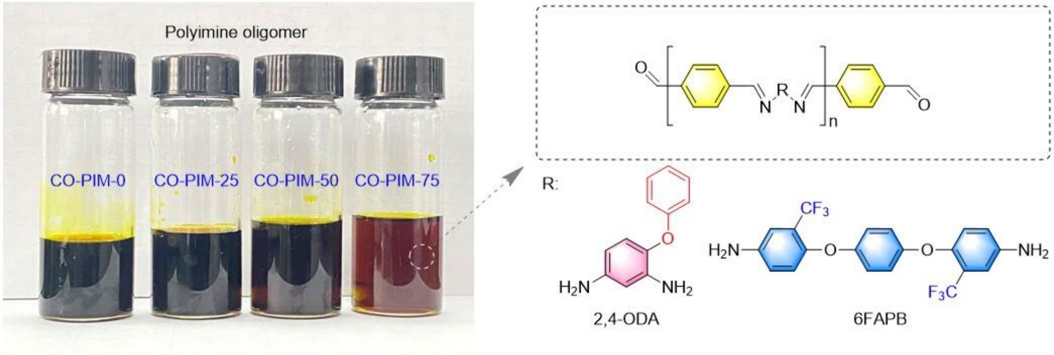

Figure S2. Photographs and chemical structures of polyimine oligomers for preparing CO-PIMs.

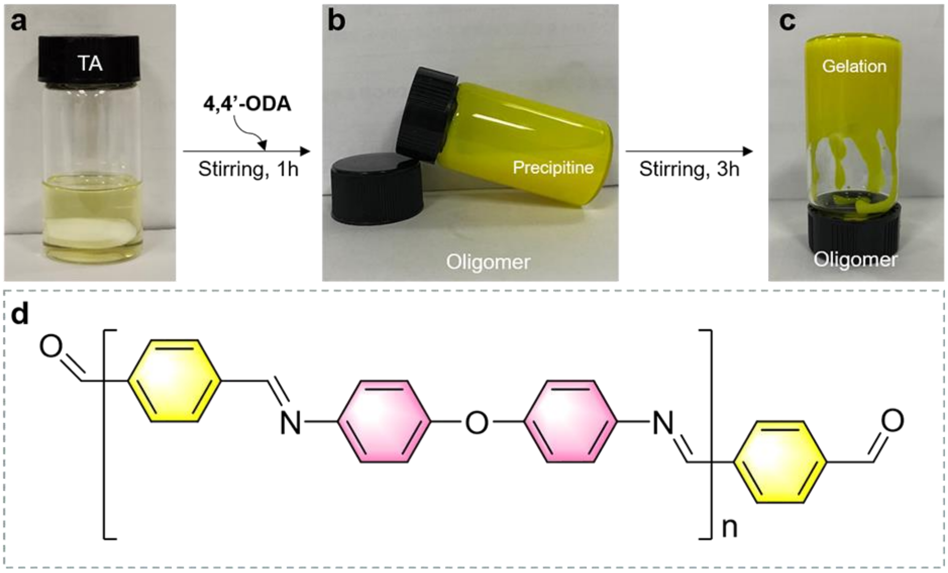

Figure S3. Photographs and chemical structures of aldehyde group terminated polyimine oligomer by using 4, 4'-ODA monomer.

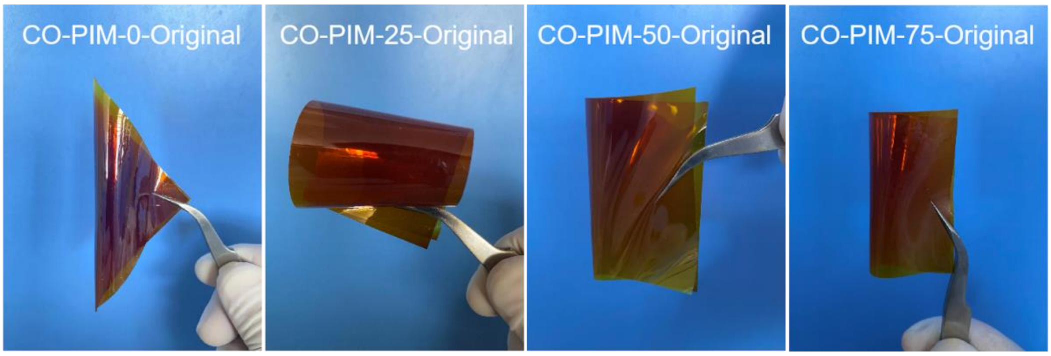

Figure S4. Images to show the films (thickness: ~0.20 mm) of CO-PIMs-Original.

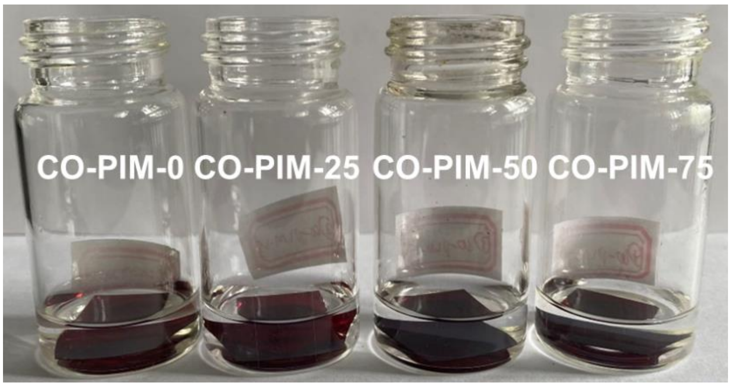

Figure S5. Digital photographs of swelling ratios and gelation content experiments.

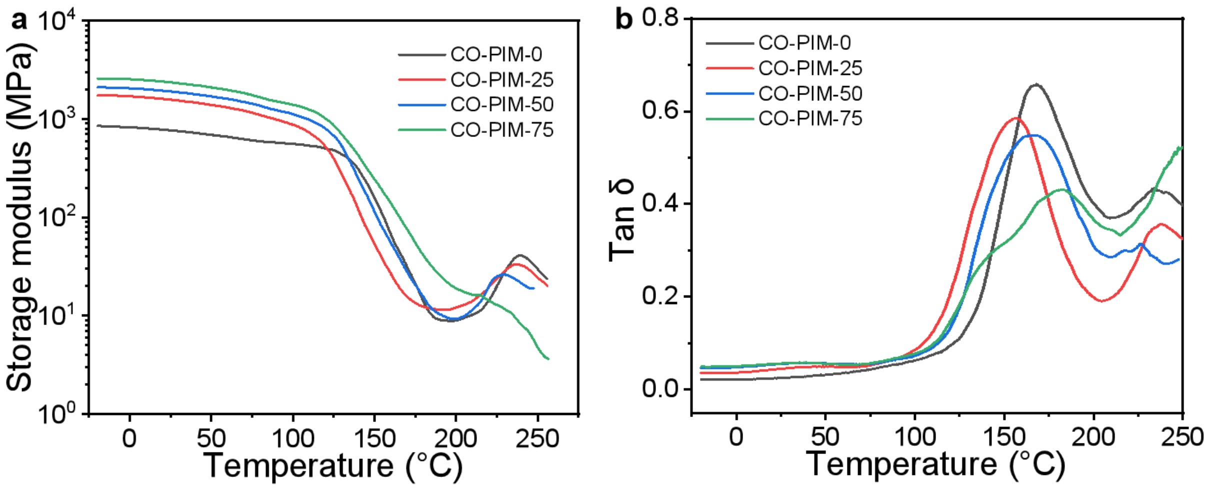

Figure S6. DMA results of CO-PIMs networks. (a) Storage modulus. (b) Loss factor (tan δ).

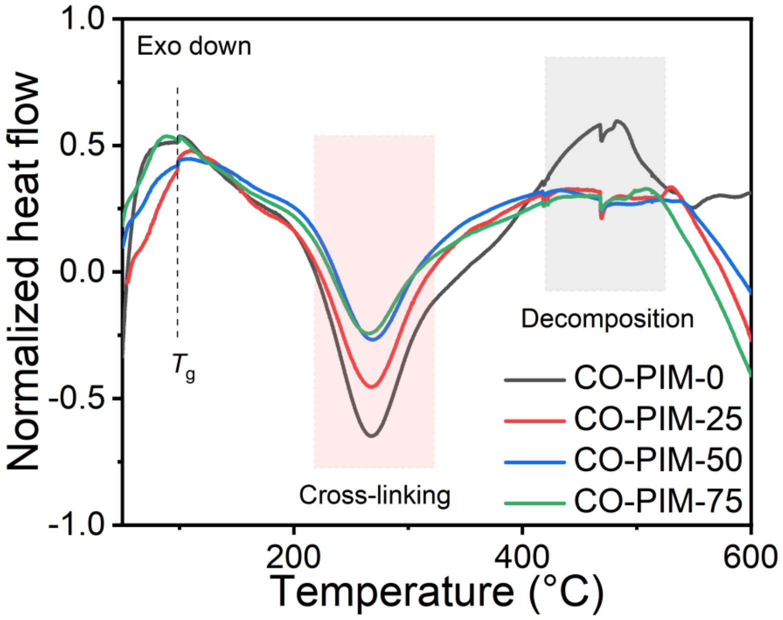

Figure S7. DSC curves of CO-PIMs recorded under TG-DSC thermograms and N_2 flow with a heating rate of 20 °C/min.

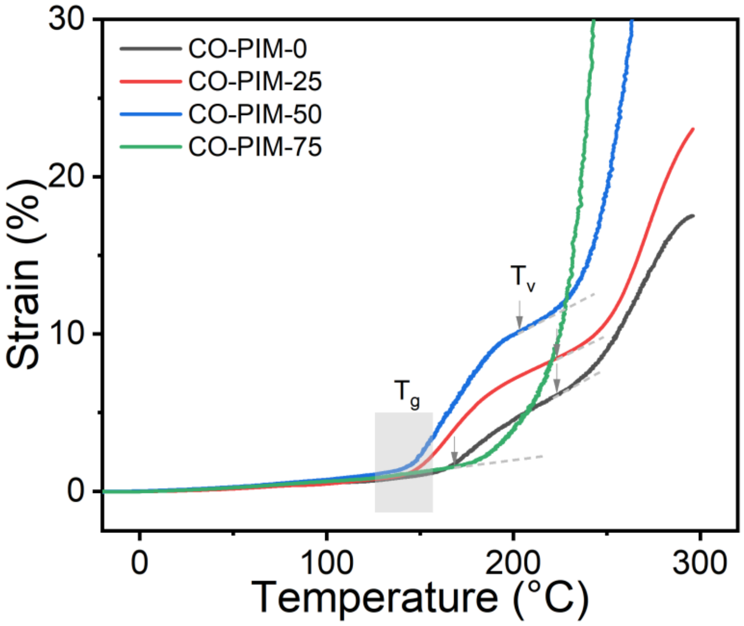

Figure S8. Temperature dependence of thermal expansion of all CO-PIMs networks with a heating rate of 5 °C/min.

Table S2. Data of thermal properties of CO-PIMs materials.

Compounds	T_g (°C) by DSC	T_g (°C) by TG-DSC	$T_g{}^a$ (°C) by DMA	ν_e (mol/m^3)	T_{d5} (°C)	T_{d10} (°C)	R_{800} (°C)
**CO-PIM-0**	53	99	168	833.3	256	306	44.8
**CO-PIM-25**	61	98	155	987.5	260	309	43.7
**CO-PIM-50**	59	99	168	897.4	244	297	42.2
**CO-PIM-75**	67	100	182	1214.6	242	299	41.3

a: regarding the peak temperature in the tanδ curves.

Table S3. Data of mechanical properties of CO-PIMs materials.

Compounds	Stress (MPa)	Elastic modulus (MPa)	Strain (%)
**CO-PIM-0**	51.6±2.2	1049.8±11.3	7.76±0.31
**CO-PIM-25**	62.1±1.9	1130.4±10.6	9.64±0.56

**CO-PIM-50**	63.7±1.5	1203.7±15.6	8.36±1.08
**CO-PIM-75**	62.5±1.7	1136.2±14.6	12.93±0.79

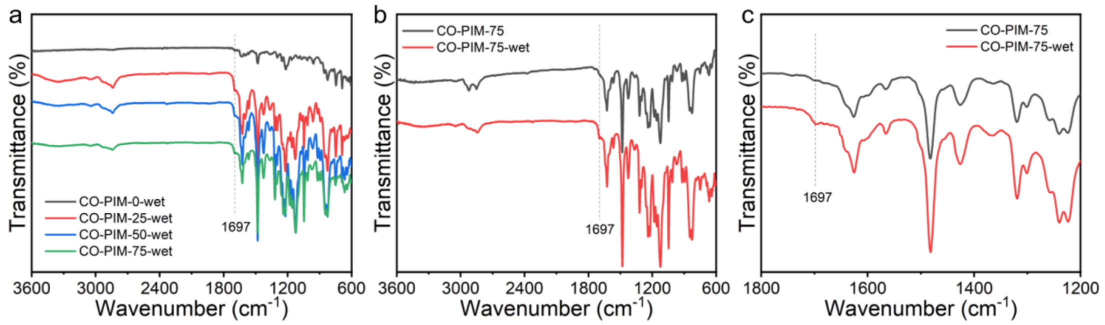

Figure S9. ATR-FTIR spectra of CO-PIMs with immersing in deionized water for 24 h (CO-PIMs-wet). a) ATR-FTIR spectra of CO-PIMs-wet. b) Comparison on the CO-PIM-75 and CO-PIM-75-wet with wavenumbers ranging from 600 to 3600 cm^{-1}. c) Comparison on the CO-PIM-75 and CO-PIM-75-wet with wavenumbers ranging from 1200 to 1800 cm^{-1}.

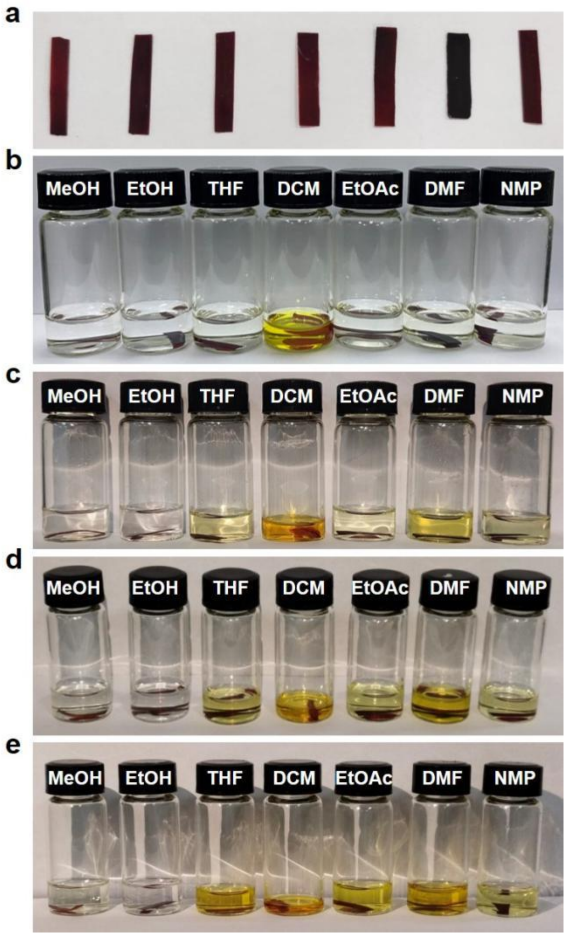

Figure S10. Digital photographs of our CO-PIM-75 samples after different time of immersion of different organic solvents: a) CO-PIM-75 samples, b) after 2 h, c) after 24 h, d) after 3 days and e) after 7 days.

Table S4. Mass change ratio of CO-PIM-75 before and after soaking in organic solvents for 7 days at room temperature.

Solvents	Mass before soaking (mg)	Mass after soaking (mg)	Mass change ratio (*D*%)
MeOH	10.2	12.3	+20.6%
EtOH	9.3	9.6	+3.2%
THF	11.1	9.7	-12.6%
DCM	12.6	6.2	-50.8%
EtOAc	19.3	17.4	-9.8%
DMF	18.4	25.3	+37.5%
NMP	10.0	10.2	+2.0%

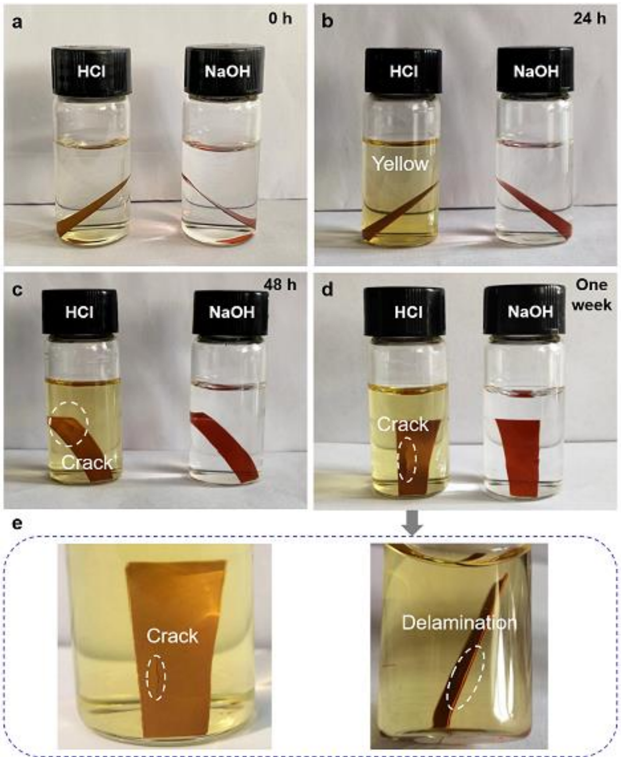

Figure S11. Digital photographs of our CO-PIMs after different days of immersion in 0.1 M acid and 0.1 M alkali. a) 0 h, b) 24 h, c) 48 h, d) one week and e) one week (enlarged view).

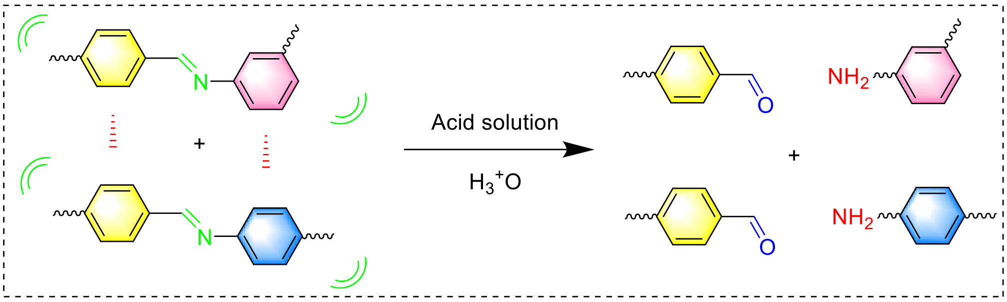

Figure S12. Degradation mechanism of our CO-PIMs with conjugated structures immersing in acid solution.

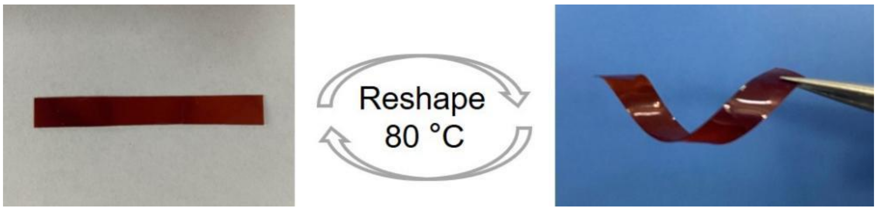

Figure S13. Digital photographs to show multiple heat-driven reshaping of an intrinsic CO-PIM-75 strip.

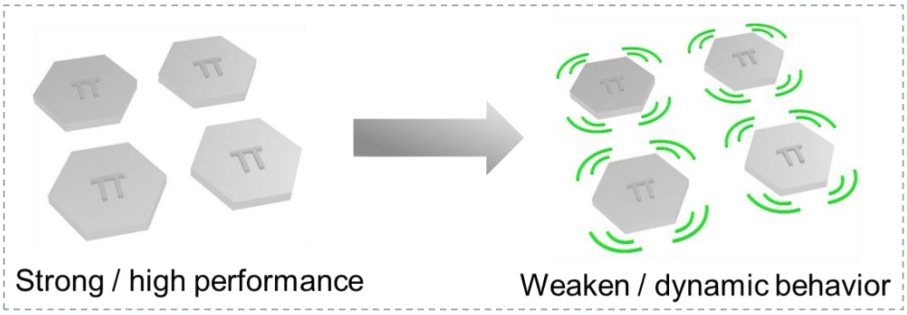

Figure S14. Mechanism diagram of molecular interaction of CO-PIMs.

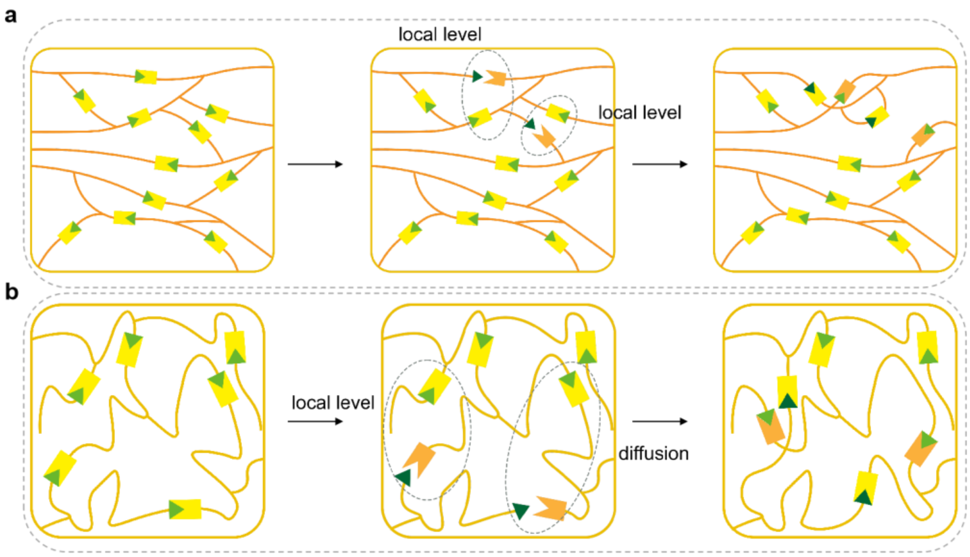

Figure S15. The activation mechanism on the introduction of trifluoromethyl diphenoxybenzene dackbones for dynamic exchange. a) rigid chains; b) flexible chains.

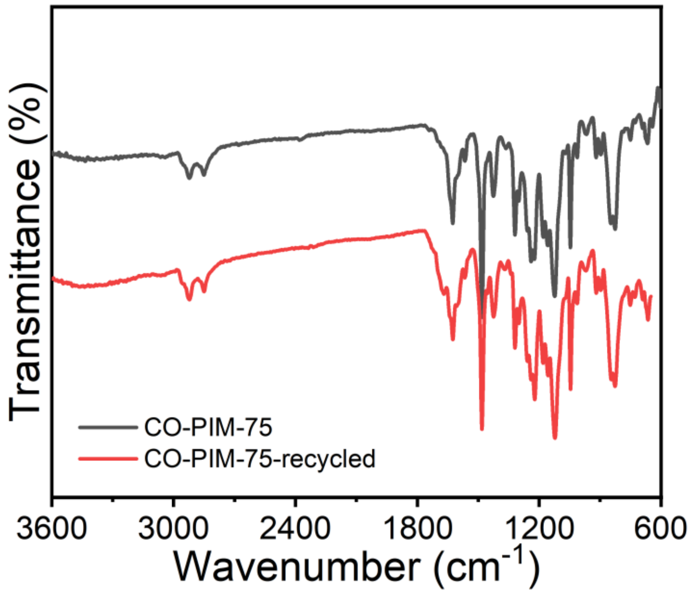

Figure S16. Typical ATR-FTIR curves of virgin and recycled CO-PIM-75 specimens.

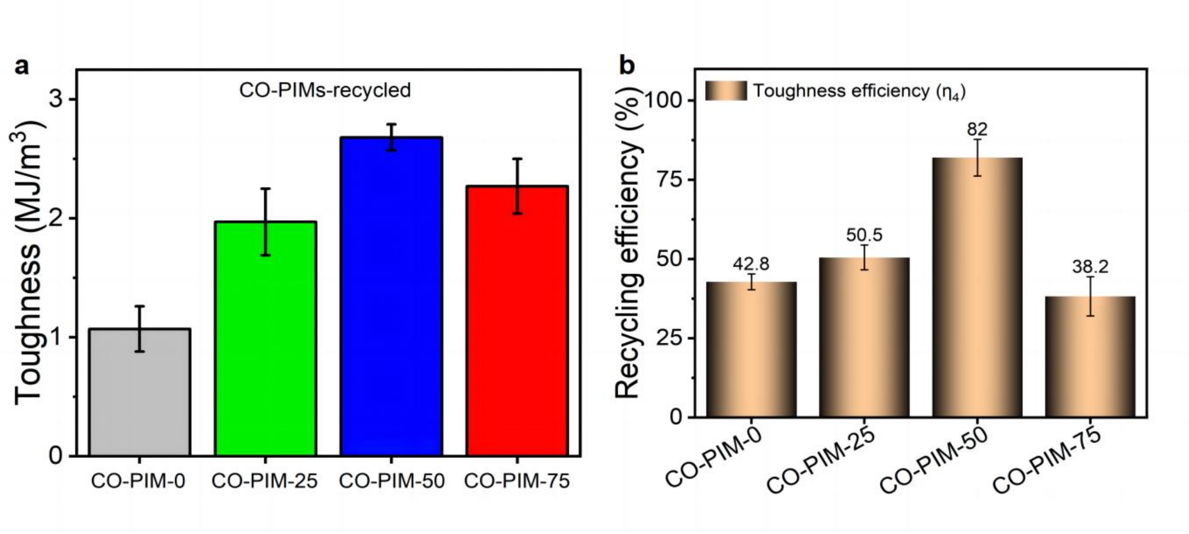

Figure S17. a) Toughness of the thermal recycled CO-PIMs specimens. b) Toughness recycling efficiency (η_4) of CO-PIMs.

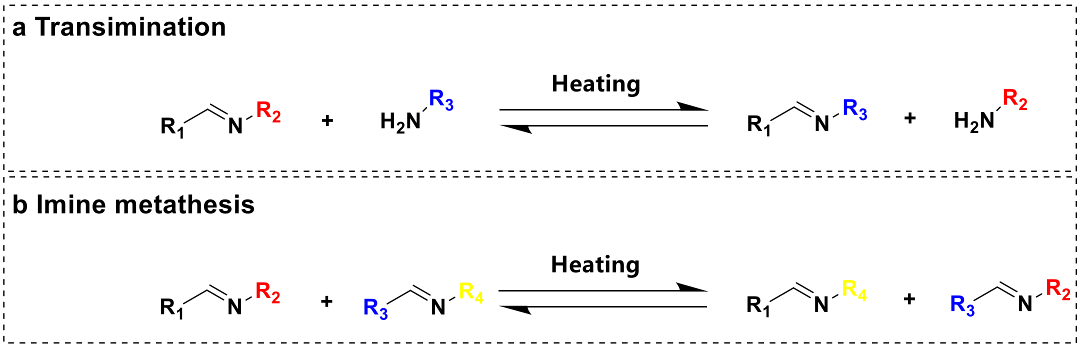

Figure S18. The mechanism of reversible exchange reactions of CO-PIMs with imine bonds.

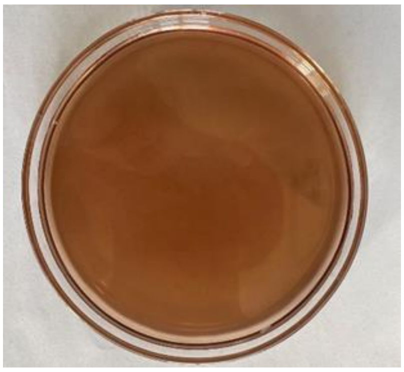

Figure S19. The free amine solution containing 2, 4-ODA, 6FAPB and NMP.

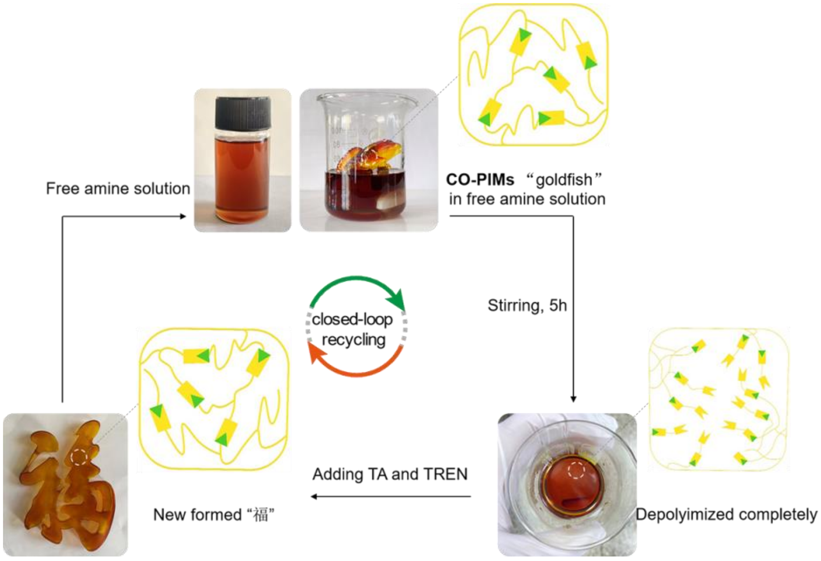

Figure S20. Schematic representation of the recycling process of CO-PIM-75 as well as the mechanism of transamination of the imine groups.

References:

[S1] J. Wu, L. Chen, T. Fu, H. Zhao, D. Guo, X. Wang, Y. Wang, *Chem. Eng. J.* **2018**, *336*, 622.

[S2] a) B. Qin, S. Liu, Z. Huang, L. Zeng, J. Xu, X. Zhang, *Nat. Commun.* **2022**, *13*, 7595; b) C. Wang, F. Eisenreich, Ž. Tomović, *Adv. Mater.* **2023**, *35*, 2209003.

[S3] M. Lyu, Y. Liu, X. Yang, D. Liang, Y. Wang, X. Liang, Y. Hu, L. Liang, C. Zhang, *Compos. Part B Eng.* **2023**, *250*, 110462.

[S4] Y. Chen, Y. Cheng, S.-P. Rwei, *ACS Sustain. Chem. Eng.* **2022**, *10*, 14794.

[S5] a) M. Capelot, M.-M. Unterlass, F. Tournilhac, L. Leibler, *ACS Macro Lett.* **2012**, *1*, 789; b) G. Lopez, L. Granado, G. Coquil, A. Lárez-Sosa, N. Louvain, B. Améduri, *Macromolecules* **2019**, *52*, 2148.

[S6] a) Z. Zhang, D. Lei, C. Zhang, Z. Wang, Y. Jin, W. Zhang, X. Liu, J. Sun, *Adv. Mater.* **2023**, *35*, 2208619; b) X. Yang, L. Cheng, Z. Zhang, J. Zhao, R. Bai, Z. Guo, W. Yu, X. Yan, *Nat. Commun.* **2022**, *13*, 6654.

[S7] P. Taynton, C. Zhu, S. Loob, R. Shoemaker, J. Pritchard, Y. Jin, W. Zhang, *Polym. Chem.* **2016**, *7*, 7052.

[S8] Z. Yang, Y. Zhang, S. Li, X. Zhang, T. Wang, Q. Wang, *ACS Sustainable Chem. Eng.* **2020**, *8*, 18869.

[S9] a) J. Zhao, Z. Zhang, L. Cheng, R. Bai, D. Zhao, Y. Wang, W. Yu, X. Yan, *J. Am. Chem. Soc.* **2021**, *144*, 872; b) Y. Ke, X. Yang, Q. Chen, J. Xue, Z. Song, Y. Zhang, S,-A. Madbouly, Y. Luo, M. Li, Q. Wang, C. Zhang, *ACS Appl. Polym. Mater.* **2021**, *3*, 3082; c) G. Li, J. Zhao, Z. Zhang, X. Zhao, L. Cheng, Y. Liu, Z. Guo, W. Yu, X. Yan, *Angew. Chem. Int. Ed.* **2022**, *61*, e202210078. d) S. Wang, S. Ma, L. Cao, Q. Li, Q. Ji, J. Huang, N. Lu, X. Xu, Y. Liu, J. Zhu, *J. Mater. Chem. C* **2020**, *8*, 11681.

[S10] X. Zhang, J. Zhao, K. Liu, G. Li, D. Zhao, Z. Zhang, J. Wan, X. Yang, R. Bai, Y. Wang, W. Zhang, X. Yan, *Natl. Sci. Rev.* **2022**, *9*, nwac012.

[S11] P. Yu, H. Wang, Y. Wang, D. Liu, Y. Xin, R. Li, X. Jia, L. Liu, D. Zhang, C. Wang, J. Zhao, Z. Zhang, X. Yan, *Chem. Eur. J.* **2023**, e202203560.

[S12] B. Wan, M. Zheng, X. Yang, X. Dong, Y. Li, Y. Mai, G. Chen, J. Zha, *Engery Environ. Mater.* **2023**, *6*, e12427.
